# Modulation of Interleukin-1 Transcriptional Response by the Interaction between VRK2 and the JIP1 Scaffold Protein

**DOI:** 10.1371/journal.pone.0001660

**Published:** 2008-02-20

**Authors:** Sandra Blanco, Marta Sanz-García, Claudio R. Santos, Pedro A. Lazo

**Affiliations:** Programa de Oncología Traslacional, Instituto de Biología Molecular y Celular del Cáncer, Consejo Superior de Investigaciones Científicas (CSIC), Universidad de Salamanca, Salamanca, Spain; University of Oldenburg, Germany

## Abstract

**Background:**

Cellular biological responses to specific stimulation are determined by a balance among signaling pathways. Protein interactions are likely to modulate these pathways. Vaccinia-related kinase-2 (VRK2) is a novel human kinase that can modulate different signaling pathways.

**Principal Findings:**

We report that *in vivo*, the activity of JIP1-JNK complexes is downregulated by VRK2 in response to interleukin-1β. Also the reduction of endogenous VRK2 with shRNA increases the transcriptional response to IL-1β. The JIP1 scaffold protein assembles three consecutive members of a given MAPK pathway forming signaling complexes and their signal can be modulated by interactions with regulatory proteins that remain to be identified. Knocking-down JIP1 with siRNA resulted in elimination of the AP1 transcriptional response to IL-1β. VRK2, a member of novel Ser-Thr kinase family, is able to stably interact with JIP1, TAK1 and MKK7, but not JNK, and can be isolated forming oligomeric complexes with different proportions of TAK1, MKK7β1 and JNK. JIP1 assembles all these proteins in an oligomeric signalosome. VRK2 binding to the JIP1 signalosome prevents the association of JNK and results in a reduction in its phosphorylation and downregulation of AP1-dependent transcription.

**Conclusions/Significance:**

This work suggests that the intracellular level of VRK2 protein can modulate the flow through a signaling pathway and alter the response from a receptor that can be distributed by more than one pathway, and thus contribute to the cellular specificity of the response by forming alternative signaling complexes. Furthermore, the effect might be more general and affect other signaling routes assembled on the JIP1 scaffold protein for which a model is proposed.

## Introduction

The cell response to specific stimulation, such as interleukins, may be transmitted by more than one signaling pathway, as is the case with interleukin-1 (IL-1) that regulates multiple biological effects [1]–[4]. In this context the modular assembly of different components of signaling pathways permits the possibility to distribute the signal among them, and depending on the interactions of the module with other proteins not only the flux, but also the subcellular localization, might be controlled [5], [6]. Among the most characterized signaling pathways are those of mitogen-associated protein kinases (MAPK), by which three different and consecutive kinases channel the response initiated at a large number of receptors in the membrane to transcription factors in the nucleus [7], [8], whose biological effects range from mitogenic to growth inhibitory responses [9]–[12], and cell survival or apoptosis [10], [13]. For each individual step there are several possible kinases thus increasing the diversity and specificity of the signal [14]–[16]. These kinases can be further modulated by their assembly with scaffold proteins [6], [17] of which the best known are the JIP protein family [18], thus contributing to achieve the specificity of particular biological effects, depending on cell type [6]. JIP1 interacts with upstream components of the c-Jun pathway in the cytosol, specifically JNK, MKK7 and some members of the MLK family [19], [20], and has been implicated in the cell response to oxidative stress [21], [22]; the regulation of apoptosis in neural cells[23], [24]; the response to some cytokines, such as IL-1β or TNF-α [1]; and with pathological conditions such as Alzheimer disease [2], [3] and type 2 diabetes [4]. The balances between positive and negative responses determine the biological effects induced by this cytokine [25], [26]. Recently modulation of signaling cascades with other interacting proteins is acquiring more relevance, as is the case in the STAT pathway [27] and in NFAT responses [28].

In the kinome there is a novel family of serine-threonine kinases composed by three members, the VRK (vaccinia-related kinase) [29], which are likely to have important biological roles in cellular signaling. These kinases are expressed in many cell types [30], [31], but their integration in new signaling pathways, or their effect in the context of known pathways are not yet known. Thus VRK1, the better known member [32], appears to be implicated in the cellular response to cellular stress based on the nature of its substrates, p53 [31], [33] forming an autoregulatory circuit [34], transcription factors ATF-2 [35] and c-Jun [36], and Baf [37], [38] required for nuclear envelope assembly. The VRK2 gene can generate by alternative splicing two isoforms, A and B, of 508 and 397 aminoacids respectively that differ in their C-terminal region. The VRK2A isoform, expressed in all cell types, has a C-terminal hydrophobic tail that anchors it to the endoplasmic reticulum and mitochondria [39], [40]. The rare VRK2B isoform, which can also stabilize p53 [40], lacks the membrane-anchor region and is detected in both cytosol and nucleus. VRK2B expression is very restricted to cell types in which VRK1 is cytosolic and thus functionally replaces it in the nucleus [40]. VRK3, another member of this kinase family, inactivates ERK signaling by a mechanism independent of its kinase activity but dependent on protein-protein interactions, promoting the interaction between ERK and its specific phosphatase VHR [41]

In this report we have studied the role of VRK2 in JNK pathway in response to IL-1β, which implicates the activation of c-Jun transcription factor [42], [43] via TAK1 [43]–[46]. We show that an interaction between VRK2 proteins and the JIP1 scaffold can modulate JNK signaling in response to IL-1β by modifying MAP kinase complexes. VRK2 protein stably interacts with the JIP1 scaffold protein and TAK1 downregulating the signals transmitted by JNK, without affecting the interaction with the other kinases of the complex, which can have wider implications in the context of cell responses mediated by MAP kinases.

## Results

### VRK2 downregulates the activation of transcription induced by IL-1β signal

**Figure 1:**
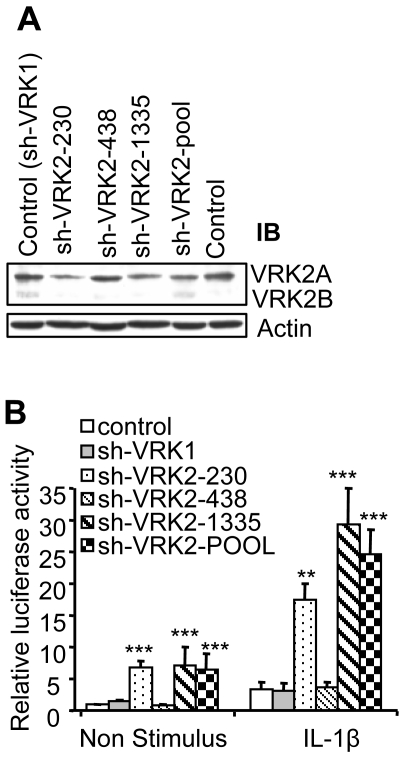
VRK2 levels modulate the transcriptional response to IL-1β. (A). HeLa cells were transfected with three shRNA constructs specific for human VRK2 or the mixture of them (sh-VKR2-pool), cloned in plasmid pSuperior-Retro-puro and targeting the indicated positions. As control a shRNA construct for VRK1 cloned in pSuperior-Retro-puro was used. Then the cells were processed for Western blotting with a specific polyclonal antibody against human VRK2 which recognizes both VRK2A (upper band) and VRK2B (lower band) isoforms. The initial level of VRK2 proteins in non-transfected cells is also shown (control). (B). Effect of shVRK2 on the transcriptional response to IL-1β. HeLa cells were transfected with 0.2 µg of reporter pAP1-luc, 10 ng of pRL-tk, and 6 µg of the plasmids expressing shRNA constructs. Four hours after transfection the cells were maintained in DMEM with 0% FBS to reduce background activity and at 40 hours the cultures were stimulated with 10 ng/ml of IL-1β (right side), or without IL-1β (left side). Extracts were collected six hours after stimulation processed for dual luciferase determination. The results from six experiments are shown. ** P<0.001, *** P<0.0005.

To determine if VRK2 proteins can have an effect on the cellular response to interleukin-1β (IL-1β) an initial approach based on RNAi was used to analyze if the change in intracellular levels of human VRK2 protein could modify the cell response to IL-1β. For this aim three shRNA specific for human VRK2 were made, but only two of them could reduce the endogenous VRK2 protein, both the common A and the rare B isoforms, in HeLa cells (Fig. 1A). Next it was determined if the transcriptional response of HeLa cells to IL-1β was affected by the VRK2 shRNA using the, pAP1-Luc reporter plasmid that responds to c-Jun or ATF2 activation by homo or hetero-dimerization [36]. In non-stimulated cells the two shRNA for VRK2, p-shRNA-VRK2-230 and p-shRNA-VRK2-1335, that induced a reduction in protein level, were also able to increase the basal level of the AP1-dependent transcription (Fig. 1B, left). If these cells were stimulated with IL-1β, there was a very significant additional increase in AP1 transcriptional activity (Fig. 1B, right), which is consistent with the reduction in both VRK2A and B protein level. While when plasmids p-shRNA-VRK1, specific for the closely related VRK1 that is highly effective in downregulating the level of endogenous VRK1 protein [31] or p-shRNA-VRK2-438 which does not alter VRK2 protein levels [31] were used (Fig. 1A), the level of activity was not affected. These results suggest that the VRK2 protein levels affect the cellular response to IL-1β, since downregulation of endogenous VRK2 expression increases the transcriptional response of AP1 to IL-1β; and suggest that the protein VRK2 functions as an inhibitor of this response since its removal results in increased transcription.

### VRK2 downregulates signals transmitted by MAP kinases

**Figure 2:**
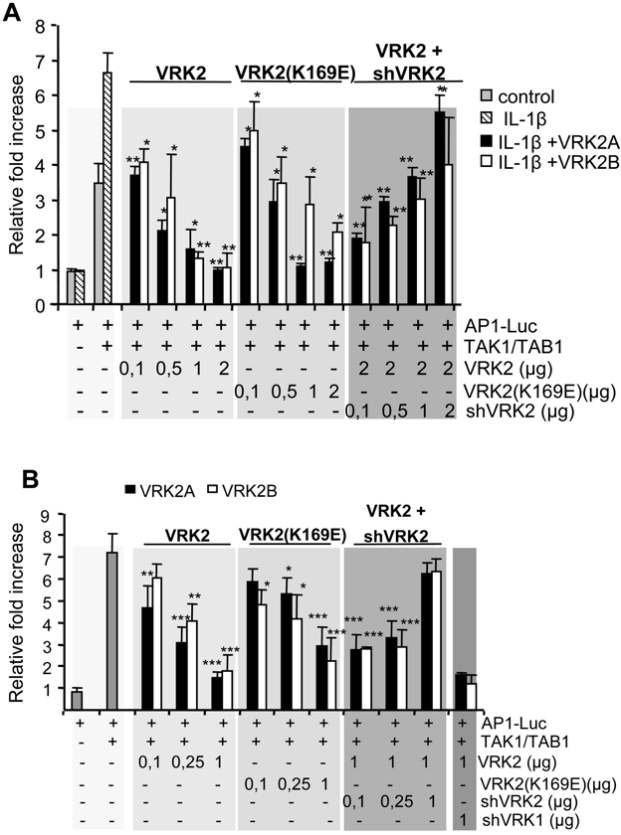
VRK2 levels effect on transcription induced by TAK1. (A). Exogenous VRK2 protein level modulates the activation of AP1-dependent transcription induced by IL-1β. The dose response effect of the wild type VRK2 proteins, the kinase-dead VRK2 proteins (K169E), or the recovery of the response by shRNA specific for VRK2 was studied in the activation of transcription by IL-1β. Cos1 cells were transfected with 0.8 µg of pAP1-luc, 10 ng of pRL-tk, pHA-TAK1 (50 ng)/pFlag-TAB1 (50 ng), and the indicated amounts of the corresponding wild-type kinase (pCEFL-HA-VRK2A or pCEFL-HA-VRK2B) or kinase dead (pCEFL-HA-VRK2A/B(K169E). The shRNA was induced using the indicated amounts of p-Superior-shRNA-VRK2-230 plasmid. 24 hours after transfection the cells were placed in serum-free media and IL-1β (10 ng/ml) were added to the culture for six hours. The results are the mean of six experiments. * P<0.05, ** P<0.005. The activity in the presence of IL-1β and TAK1/TAB1 and in the absence of any VRK2 plasmid was used as reference (First shaded box) for statistical analysis. (B). Effect of VRK2 levels on AP1-dependent transcription mediated by TAK1 activation of the endogenous JIP1-JNK signaling complex. Cos1 cells were transfected with 0.8 µg of the reporter pAP1-Luc plasmid, 10 ng of pRL-tk, pHA-TAK1 (50 ng) and pFlag-TAB1 (50 ng) and the indicated amount of plasmids expressing the VRK2 isoforms, active (pCEFL-HA-VRK2A or pCEFL-HA-VRK2B) or kinase-dead variants (with the K169E substitution) and plasmid pSuperior-shRNA-VRK2-230 for RNA interference. The correct expression of the proteins was first determined by immunoblot analysis (not shown). The value used as reference was the maximal activity obtained when stimulated with TAK1/TAB1. The results from six experiments are shown. * P<0.05, ** P<0.005, *** P<0.0005. The minor differences between VRK2A and VRK2A(K169E) were not statistically significant. The activity in the presence of TAK1/TAB1 and absence of any VRK2 plasmid was used as reference for statistical analysis.

Transcriptional responses induced by IL-1β, and mediated by AP1 sites, require the activation of JNK via TAK1 [43], [44], [47]–[50]. Therefore, in order to determine if VRK2 levels, its kinase activity or the different isoforms might modulate TAK1 pathway in response to IL-1β response pathway, Cos1 cells were transfected with the pAP1-Luc reporter, and plasmids expressing TAK1 and its cofactor TAB1 [49] in order to restrict the IL-1β response to a unique MAPK pathway. Cos1 cells were treated with or without IL-1β, but in the absence of TAK1/TAB1 overexpression, AP1 dependent transcription were not stimulated with IL-1β (Fig. 2A, first shaded box) because these cells do not express TAK1 [51]. TAK1/TAB1 by itself activated AP1 dependent transcription that was further increased if cells were stimulated with IL-1β (Fig. 2A, first box). The cotransfection with VRK2A, VRK2B (Fig. 2A, second box), or their kinase-dead (K169E substitution), variants that contain the K169E substitution in the catalytic site [40] (Fig. 2A, third box) resulted in a significant reduction of the transcriptional response to IL-1β, which was dose dependent, and consistent with the inhibitory role proposed for VRK2 in the previous section. To confirm the dependence of the AP1 transcriptional downregulation on the level of VRK2 proteins, an experiment using specific shRNA was designed. The point of maximum effect induced by VRK2A and VRK2B was selected to analyze the consequence of shRNA with plasmid p-shRNA-VRK2-230. The increase in the shRNA specific for VRK2 was able to restore the induction of transcription by TAK1/TAB1 (Fig. 2A, fourth shaded box). These observations suggested that VRK2 proteins, independently of their activity or the isoform used, but in a dose dependent manner, were able to interfere with the signal generated in response to IL-1β, and mediated by the TAK1 pathway.

Next we attempted to establish at what stage, between the receptor and the transcription factor, was VRK2 acting in the IL-1β response pathway. For this aim, a similar assay was used, but the endogenous MAPK pathway was stimulated only by overexpression of the active form of TAK1 with TAB1. TAK1/TAB1 strongly activated the transcription mediated by the AP1 response element (Fig. 2B, first shaded box). Increasing amounts of either VRK2A (black bars) or VRK2B (white bars) resulted in a significant downregulation of the TAK1/TAB1 activation of transcription (Fig. 2B, second shaded box). The kinase-dead, VRK2 (K169E) proteins similarly induced a downregulation of the activation of transcription (Fig. 2B, third shaded box). The negative effect on transcription induced by the maximum amount of VRK2A or VRK2B was reversible in a dose dependent manner using p-sh-RNA-VRK2-230 plasmid, specific for human VRK2 (Fig. 2B, fourth box). The shRNA specific for the closely related VRK1 was used as before as a negative control and had no effect (Fig. 2B, fifth shaded box). These results indicate that VRK2 interferes with the IL-1β signal at the MAP kinase level. And the most likely explanation for these results is by a physical interaction of VRK2 with some component of the signaling pathway, located between TAK1 and the activation of the transcription factor.

### VRK2 stably interacts with JIP1

**Figure 3:**
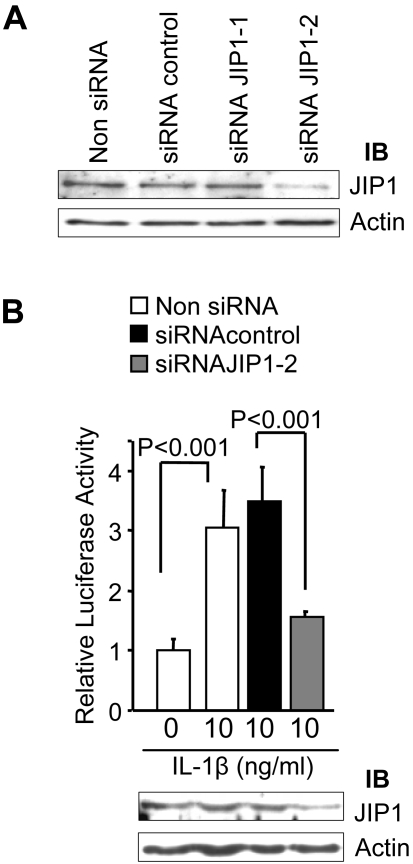
JIP1 levels modulate the transcriptional response to IL-1β. (A). Effect of siRNA specific for JIP1 on its level. HeLa cells were transfected with two different siRNA specific for JIP1; 48 hours after transfection the level of endogenous JIP1 protein was determined with a specific antibody. The siRNA-2, but not siRNA-1, was able to knock down JIP1 protein levels. (B) Effect of siRNA for JIP1 on the transcriptional response to IL-1β. HeLa cells were firstly transfected with 100 pmoles of siRNA-2 for JIP1 and 100 pmoles of siRNA control as indicated. 24 hours after, cells were retransfected with 0.2 µg of reporter pAP1-luc and 10 ng of pRL-tk. Four hours after the second transfection the cells were maintained in DMEM with 0% FBS to reduce background activity and at 40 hours the cultures were stimulated with 10 ng/ml of IL-1β, or without IL-1β. Extracts were collected six hours after stimulation and processed for dual luciferase determination (top) and western blot analysis of JIP1 levels (bottom). Results are the means of three experiments. Blots were quantified in the linear response range.

The effect of VRK2 is independent of its kinase activity; therefore it is likely to be mediated by protein-protein interactions with MAPK kinase complexes formed in response to IL-1β signaling. One likely candidate is the scaffold protein JIP1, which assembles and regulates the MAP kinases of the JNK signal transduction pathway MLK3, MKK7 and JNK [20], [52]–[54], and is required for JNK activation in response to cytokine stimulation such as IL-1β but unnecessary for JNK activation induced by UV radiation or anisomycin [1], [20], [55]. In order to confirm that JIP1 is necessary for JNK activation in response to IL-1β, an experiment based on RNAi silencing was performed. First two siRNA specific for JIP1 were tested (Fig. 3A). The partial reduction of endogenous JIP1 protein levels with one of the siRNA was accompanied by a proportional reduction in the transcriptional response to IL-1β mediated by AP1 (Fig. 3B), suggesting that JIP1 is a critical component in the transcriptional response to IL-1β mediated by AP1. Recently TAK1 has been shown to bind to JIP1, and its binding increases JNK association to JIP1 and activation. The complex JIP1-TAK1 is required for JNK activation in response to hypoxia [51].

**Figure 4:**
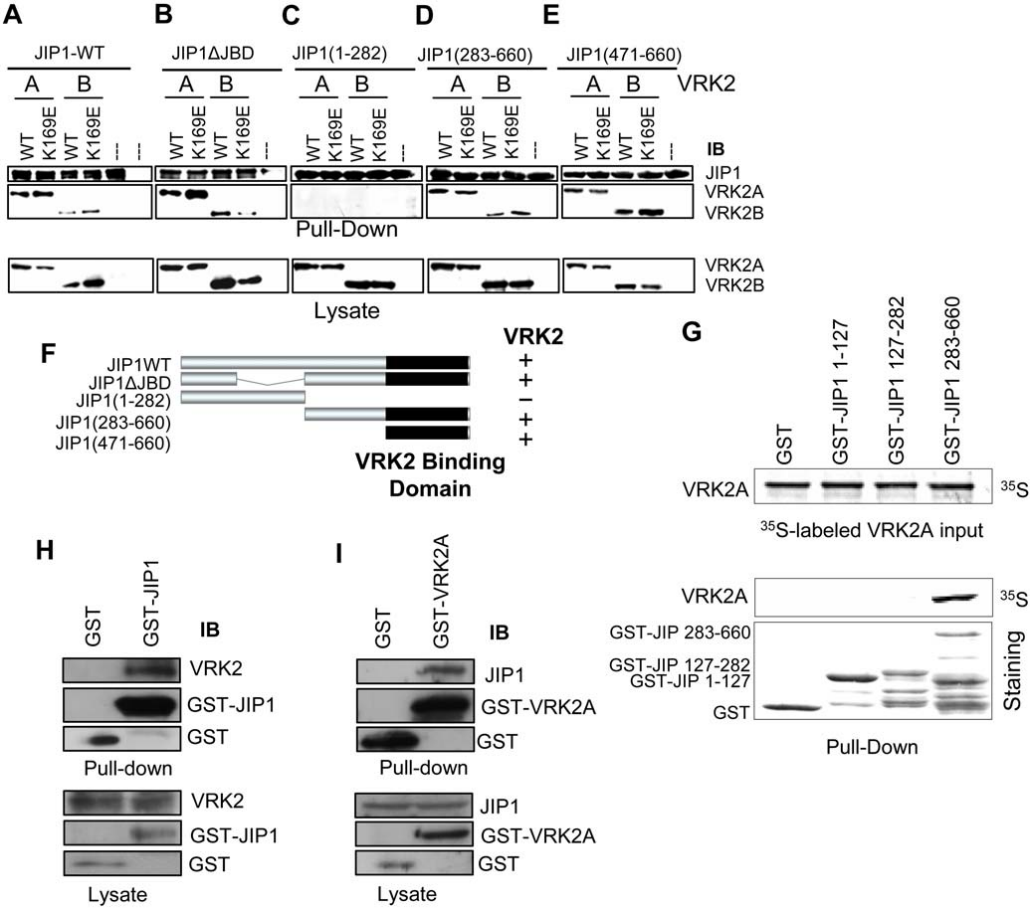
Mapping the region of JIP1 that interacts with VRK2 isoforms. (A–F). Cos1 cells were transfected with plasmids as indicated in the corresponding lane. The expression of the proteins was determined by western blot (bottom panel). The different lysates were pulled down with Glutathione-Sepharose to bring down the GST-JIP1 protein and associated molecules. The pull-down proteins were detected with antibodies that recognize the HA epitope in the VRK2 proteins. The constructs derived from VRK2 includes the two isoforms expressed from plasmids pCEFL-HA-VRK2A and pCEFL-HA-VRK2B as well as their catalytically inactive kinase-dead (KD) variants containing the K169E substitution. JIP1ΔJBD lacks the JNK binding domain (residues 127-282). (G). In vitro interaction between human VRK2 and JIP1 proteins. The human VRK2 protein was in vitro transcribed-translated and labeled with 35S methionine. This protein was used for a pulldown assay with bacterially expressed GST-JIP1 constructs. (F). Schematic representation of the GST-JIP1 proteins used in the pull-down assay and the interacting region with VRK2. (H). Binding of endogenous VRK2A to GST-JIP1. Cos1 cell were transfected with pCEFL-GST or pEBG-GST-JIP1, and the proteins in the pull-down were identified with a specific polyclonal antibody for VRK2 and GST. (I). Binding of endogenous JIP1 protein to GST-VRK2A. Cos1 cells were transfected with pCEFL-GST or PCEFL-GST-VRK2A. The JIP1 protein in the pull-down was detected with a specific polyclonal antibody.

Next it was studied the possibility that JIP1 might interact in a stable manner with other proteins, such as VRK2A or VRK2B, which in turn might modify MAPK kinase complex assembly. To address this point Cos1 cells were transfected with a mammalian construct, pGST-JIP1, expressing the GST-JIP1 full-length fusion protein or different constructs spanning parts of the JIP molecule, JIP1-ΔJBD (lacking residues 127-282, the JNK binding domain), 1-282, 283-660 and 471-660 [52]. These constructs were cotransfected in combination with plasmids pCEFL-HA-VRK2A, pCEFL-HA-VRK2B or their kinase-dead (K169E) constructs. These cell lysates were mixed with glutathione-Sepharose beads and a pull down of the GST-JIP1 protein was performed to determine the associated proteins by immunoblot analysis. Both, VRK2A and VRK2B, were able to form a stable complex with JIP1 (Fig. 4A), and the kinase activity was not necessary for the stable interaction since kinase-dead (K169E) proteins also interacted although in a stronger manner. The amino terminal region of JIP1 did not interact with VRK2 (Fig. 4C), neither the region that interacts with JNK, residues 127-282 (ΔJBD, in Fig. 4B) [20]. The minimal region of JIP1 required for interaction with VRK2 isoforms is located within residues 471 to 660 (Fig. 4E), and the interaction of this C-terminal construct GST-JIP1 (471-660) with VRK2B appeared to be stronger (Fig. 4E). Therefore we concluded that the JIP1 region implicated in the interaction with VRK2 proteins is located in its C-terminal domain between residues 471 and 660. This JIP1 region implicated in the interaction with VRK2 is different from the one required to interact with JNK or TAK1. Although the interaction is independent of the kinase activity, both VRK2 isoforms (VRK2B not shown) were able to phosphorylate the JIP1 N-terminal region, within residues 1-127 and outside its docking region for JNK. This phosphorylation might explain why the binding of VRK2 inactive mutants to JIP1 is more robust, since these kinase inactive mutants bind to the substrate but are unable to transfer the phosphate and therefore the substrate cannot be released, causing the formation of a stable intermediate [40].

To demonstrate that the interaction between VRK2A and JIP1 is direct, an in vitro interaction assay was performed. VRK2A was in vitro translated and used for a pull-down-assay using different GST-JIP1 protein constructs expressed in bacteria. VRK2A was able to interact with the C-terminal region of JIP1 (Fig. 4G) confirming that the interaction does not require any additional protein.

Next it was determined if the interaction could also be detected with the endogenous proteins. Therefore an experimental approach based on pull-down of endogenous proteins was used in a reciprocal way. The endogenous VRK2 protein was brought down in a pull-down assay with GST-JIP1 (Fig. 4H). In the reciprocal experiment, the endogenous JIP1 protein was also brought down by GST-VRK2A (Fig. 4I). These data supports the specificity of the interaction between VRK2A and JIP1.

### JIP1 colocalizes with VRK2 and is associated to endoplasmic reticulum and mitochondria

**Figure 5:**
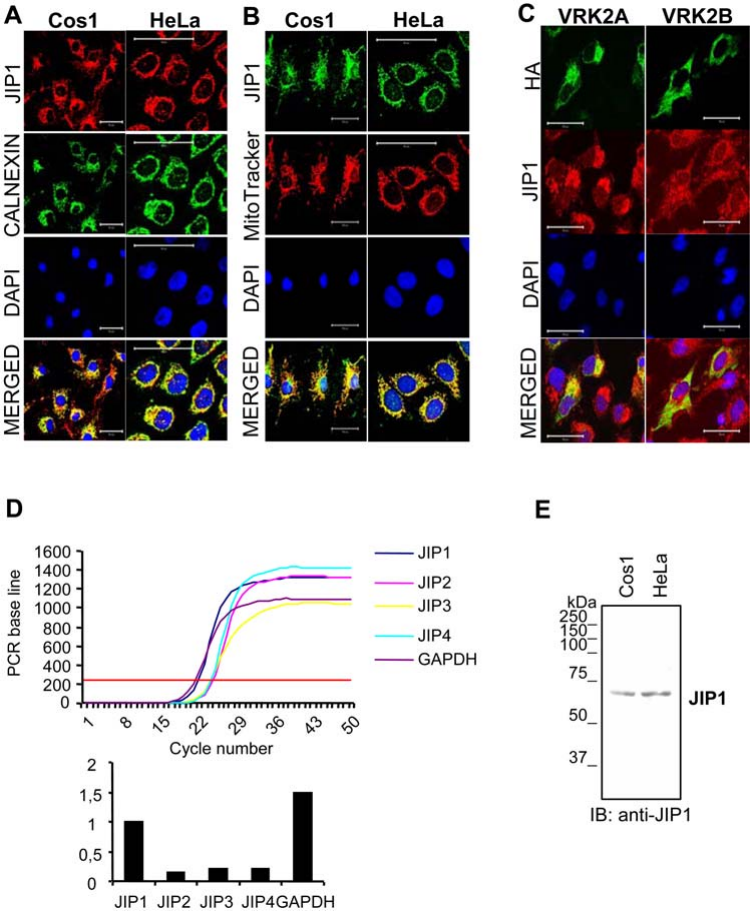
Subcellular localization of endogenous JIP1 in the endoplasmic reticulum and mitochondria protein and colocalization with VRK2A. (A). Association of endogenous JIP1 with the endoplasmic reticulum. JIP1 was detected with a rabbit polyclonal antibody and as secondary a Cy3-labeled anti-rabbit antibody was used (red). Calnexin was detected with a monoclonal antibody and a Cy-2-labeled anti-mouse antibody (green). (B) Association of endogenous JIP1 with mitochondria. JIP1 was detected with a rabbit polyclonal antibody and as secondary antibody a Cy2-labeled anti-rabbit antibody was used (green). Mitochondria were detected with the MitoTracker Red CMXRos reagent (red). Nuclei were identified with DAPI staining. The bar indicates 50 µm. (C). Colocalization of endogenous JIP1 and transfected HA-VRK2A. The Cos1 cell line was transfected with plasmid, pCEFL-HA-VRK2A and pCEFL-HA-VRK2B, expressing each of the VRK2 isoforms. The endogenous JIP1 protein was detected with a rabbit polyclonal antibody (red) and the transfected VRK2 proteins with a monoclonal antibody specific for HA epitope (green). Nuclei were identified with DAPI staining. The bar indicates 50 µm. (D). The expression of the four human JIP (1–4) genes was determined by quantitative RT-PCR in RNA extracted from HeLa cells as described in Material and Methods. The profile of amplification (upper panel) as well as the quantification (lower panel) by the Bio-Rad iCycler iQ5 Software is shown. (E). Detection of endogenous JIP1 protein in HeLa and Cos1 cells by immunoblot using a polyclonal antibody against JIP1.

To confirm the association of VRK2 and JIP1 it was analyzed the subcellular location of each molecule and the possible colocalization. JIP1 has been reported to be located in the perinuclear region with a particulate pattern in neural cells, suggesting its association with membranes such as mitochondria [56], or other organelles still unidentified [57]. These data suggests that a fraction of JIP1 protein is likely to have a subcellular location similar to VRK2. This led us first to determine the subcellular location of the endogenous JIP1 protein in two cell lines; Cos1 and HeLa (human cervical carcinoma cell line), given that JIP1 is the family member more ubiquitously expressed, although is expression is more abundant in brain, testis, lung, kidney and pancreas [20], [58]. In all of them JIP1 presented a particulate pattern (Fig. 5A, B). To determine the type of membrane, the endoplasmic reticulum (ER) was identified using an antibody specific for calnexin, and mitochondria were detected with Mitotracker reagent. The JIP1 signal colocalized, in both cell lines, indicating close proximity with both ER (Fig. 5A) and mitochondrial markers (Fig. 5B).

The subcellular location detected for endogenous JIP1 protein is very similar to that reported for the full length VRK2A protein that is anchored to membranes by its C-terminal transmembrane region [40], suggesting a potential colocalization between JIP1 and VRK2 isoforms. For this aim it was necessary to transfect Cos1 cells with pCEFL-HA-VRK2A or pCEFL-HA-VRK2B plasmids, expressing the two VRK2 isoforms that were detected with a monoclonal antibody specific for the HA epitope, since the available antibodies for endogenous proteins are both rabbit polyclonal antibodies. The pattern of endogenous JIP1 protein overlapped in part with the VRK2A signal that has been reported to be bound to the endoplasmic reticulum by its C-terminal region (Fig. 5C, left column). In the case of VRK2B the pattern detected is much more diffuse in the cytosol (Fig. 5C, right column) [40]. These data support the physical interaction between JIP1 and the membrane bound VRK2A protein.

To establish that JIP1 is the main human JIP gene expressed in HeLa cells, the expression of the four JIP genes, 1 to 4, was determined by RT-PCR. JIP1 expression is at least ten fold higher than the rest of JIP messages expressed in this cell type (Fig. 5D). This main form is recognized by the corresponding JIP antibody in both cell lines (Fig. 5E).

### VRK2A, but not VRK2B, directly interacts with TAK1

**Figure 6:**
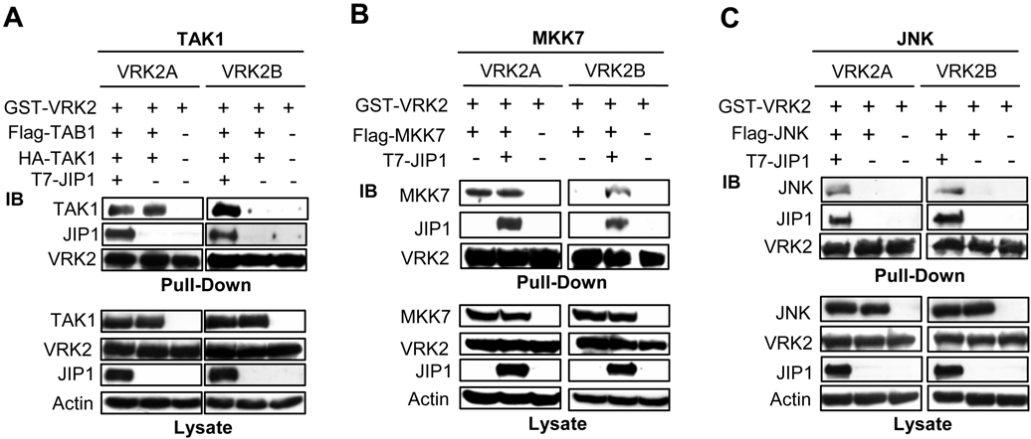
Individual VRK2 interactions with MAP kinases. (A). Interaction of TAK1/TAB1 with VRK2 isoforms. (B) Interaction between MKK7 and VRK2. (C). Interaction between JNK and VRK2. Cos1 cells were transfected with plasmids expressing the indicated proteins. pT7-JIP1(4 µg), pCMV-HA-TAK1(20 ng) plus de pCMVT-Flag-TAB1(20 ng), pCEFL-GST-VRK2A or pCEFL-GST-VRK2B(4 µg), pFlag-MKK7 (0,5 µg) or pFlag-JNK (5 µg). The pull-down and immunoblots were performed as described in the methods section and the proteins were detected with antibodies against VRK2, JIP1 and Flag and HA epitopes.

Because of the oligomeric nature of the signalosome, it was also tested if VRK2A or VRK2B could also interact with any of the three MAP kinases in the route independently of JIP1. For this aim cells were transfected with pGST-VRK2A or pGST-VRK2B, and plasmid expressing the corresponding kinase, TAK1, MKK7 or JNK with and without JIP1. In the absence of JIP1, TAK1 (Fig. 6A) [51], and MKK7 (Fig. 6B), but not JNK (Fig. 6C), were able to stably interact with VRK2A, which is the isoform expressed in most cell types. Since this cells expresses JIP1 is also possible that the interaction of TAK1 or MKK7 and VRK2A was mediated by endogenous JIP1, but in that case VRK2B also would interact with those MAP kinases, making these interactions specifics for VRK2A. In fact it is been reported that TAK1 interacts by the C-terminal domain of VRK2A which is absent in VRK2B [51]. The VRK2B isoform did not stably interact with TAK1 or MKK7 in the absence of JIP1, which explains why the binding of VRK2A with JIP1 is always stronger that the JIP1-VRK2B interaction (Fig. 6A), this difference might be explained because VRK2A also interact with these MAP kinases making the complex more stable [51]. The association of VRK2A with TAK1 and MKK7, independently of JIP1, might titer away these upstream kinases by adding increasing amounts of VRK2A, making them unavailable for JNK activation, explaining the downregulation effect caused by VRK2. However, VRK2B does not interact with TAK1 or MKK7 and therefore can not titer them away.

### Effect of VRK2 on the interaction of JIP1 with consecutive MAP kinases

**Figure 7:**
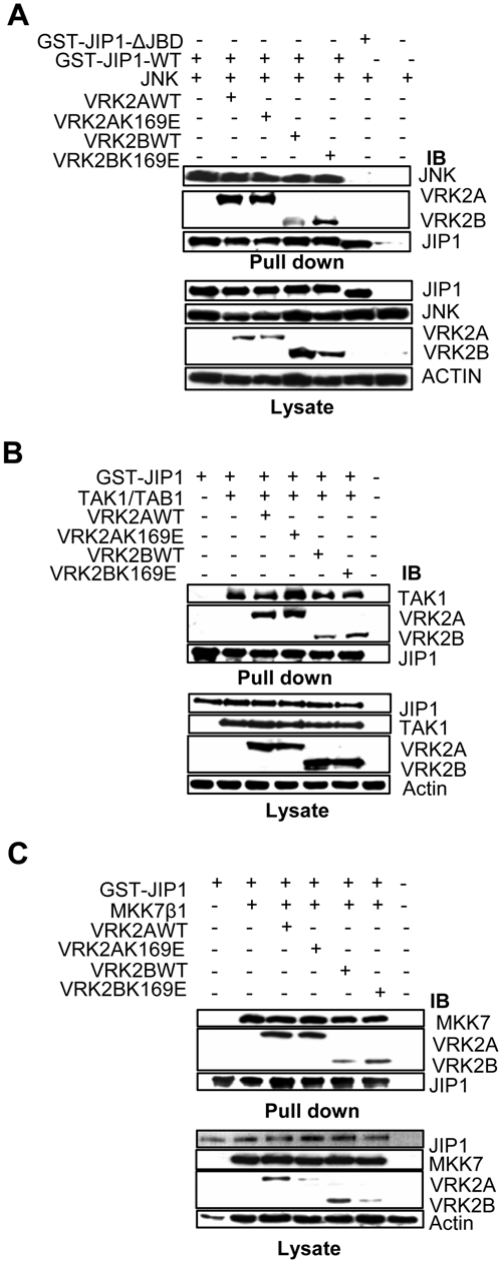
Effect of VRK2 on the interaction of JIP1 with different MAP kinases and detection of JIP1 signalosome. (A). Effect of VRK2A and B on the JIP1-JNK interaction. The plasmids used were pEBG-GST-JIP1(3 µg), pFlag-JNK(3 µg) and pCEFL-HA-VRK2A or pCEFL-HA-VRK2B wild-type or kinase-dead (5 µg). The proteins were detected with antibodies for actin and the corresponding epitopes, HA, GST, and Flag. (B). Effect of VRK2A orVRK2B on the TAK1-JIP1 interaction. The plasmid used in Cos1 cell transfections were pEBG-GST-JIP1(3 µg), pCMV-HA-TAK1(50 ng) plus de pCMVT-Flag-TAB1(50 ng) and de pCEFL-HA-VRK2A/B wild-type or kinase-dead (5 µg). The proteins were detected with antibodies for actin and the corresponding epitopes, HA, GST, and Flag. (C). Effect of VRK2A orVRK2B on the MKK7β1-JIP1 interaction. Cos1 cells were transfected with pEBG-GST-JIP1(3 µg), pFlag-MKK7β1(1 µg) and pCEFL-HA-VRK2A/B wild-type or kinase-dead(5 µg). The proteins were detected with antibodies for actin and the corresponding epitopes, HA, GST, and Flag.

The scaffold protein JIP1 assembles signaling complexes composed of three different and functionally consecutive MAP kinases, and these associations may be affected by additional interactions with proteins that are not components of the signaling cascade. Therefore it was first determined if the stable association of JIP1 with VRK2 proteins could have any effect on the binary combinations of JIP1 with some of these MAP kinases. For this aim, a kinase representing each of the three consecutive steps in the cascade was selected for analysis; TAK1 (as a MAPKKK) that is known to participate in IL-1β response [43], [44], [47]–[50], MKK7β1 (representing a MAPKK), and JNK. The experimental approach followed was similar for the three steps. Cos1 cells were cotransfected with plasmids expressing GST-JIP1, the corresponding MAP kinase, and the VRK2 protein isoforms, either wild type or kinase-dead (K169E). Once their correct expression was confirmed in the cell lysates (bottom panels of Fig. 7A, B and C), these were used for a pull-down of GST-JIP1, and then the associated proteins were analyzed by western blot. We focused on the level of each individual MAP kinase pulled down with GST-JIP1 in the absence or presence of the different VRK2 forms. In these assays the level of JNK protein bound to JIP1 did not appear to be significantly affected by either form of VRK2 since the JNK protein levels were the same in each case (Fig. 7A, upper panel). The kinase activity of VRK2A or B also did not have any effect on the JNK interaction either. We used the ΔJBD JIP1 mutant that lacks the JNK-binding domain as a negative control. Similarly, the wild type TAK1 (Fig. 7B, upper panel) or MKK7β1 (Fig. 7C, upper panel), were able to stably interact with JIP1, and this interaction did not appear to be affected by any of the VRK2 isoforms. In conclusion both VRK2 isoforms did not affect the binary combinations of the MAP kinases mentioned above with JIP1 or the effect can not be noticed when the complex is not complete or active as in this case, since only one MAP kinase has been overexpressed in each assay.

### JIP1 signalosome: assembly of an oligomeric complex

**Figure 8:**
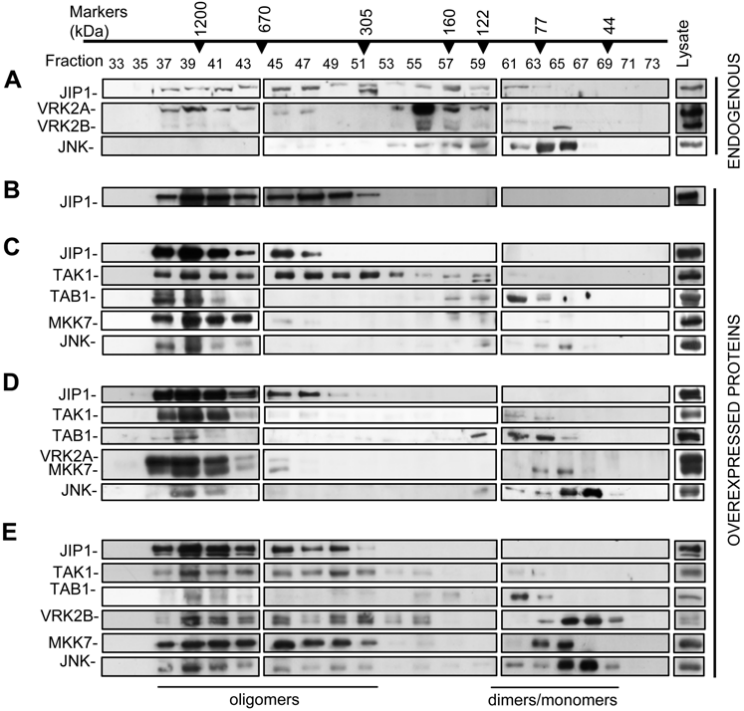
The JIP1 signalosome: oligomerization of MAP kinases and VRK2 proteins in the presence of JIP1. (A) Composition of endogenous signalosome formed by JIP1, VRK2 proteins and JNK in non transfected Cos1 cells. (B). Detection of JIP1 oligomeric complexes in Cos1 cells transfected with only 3 µg of pGST-JIP1. (C) Reconstitution of JIP1 signalosome in the absence of VRK2 proteins in Cos1 cells transfected with 3 µg of pGST-JIP1, 50 ng of pHA-TAK1, 50 ng of pFlag-TAB1, 0,2 µg of pFlag-MKK7, and 4 µg of pFlag-JNK. (D) Effects of VRK2A on the JIP1 signalosome. Cos1 cells were transfected with the plasmids indicated in C plus 4 µg of pCEFL-HA-VRK2A. (E) Effects of VRK2B on the JIP1 signalosome. Cos1 cells were transfected with the plasmids indicated in C plus 4 µg of pCEFL-HA-VRK2B. Cell extracts were fractionated in a Superose 12 10/300 GL column that fractionates proteins in the range from 50 to 1500 kDa. The fractions were analyzed in western blots with antibodies for the specific epitope in each protein. The corresponding molecular weight of the fractions is indicated above. The calculated molecular weights of each monomeric protein are; JIP1 77 kDa, VRK2A 55 kDa, VRK2B 43 kDa, TAK1 64 kDa, TAB1 55 kDa, MKK7 48 kDa and JNK1 46 kDa.

The interaction between JIP1 and VRK2 may be exclusive of JIP1-MAP kinase individual interactions since pull down experiments performed before can not discriminate between complexes formed by more than three proteins, and immunoprecipitation with antibodies might interfere or compete with binding of additional proteins, thus precipitating only the non complexed combinations available to the antibody. Therefore the different possible combination of interacting proteins, or even the formation of large complexes, was assayed when all of them are expressed at the same level. The protein complexes were separated by performing a gel filtration chromatography in a Superose 12 10/300 GL column that specifically separates native molecules ranging from 50 to 1500 kDa and permits to detect all different protein combinations present in complexes. The different fractions were analyzed in western blots to identify its components. First it was determined the complex formation of oligomeric endogenous JIP1 and VRK2 proteins in Cos1 cells. These two proteins are forming a large complexes of different sizes (Fig. 8A), but the endogenous JNK is mostly free, probably because the cells were not stimulated and therefore the complex remains in a latent state, hence JNK is not gathered to the whole complex. Some JNK is also detected in small complexes formed by two or three proteins, as is the case for most of the endogenous VRK2A protein (Fig. 8A). Surprisingly JIP1, endogenous or transfected were forming large complexes in the range 300 to 1200 kDa (Fig. 8A, B). In the case of endogenous JIP1 also smaller complexes were detected, but they contain bound endogenous VRK2A, which is not detected free (Fig. 8A). A possible explanation is that the polymerization of the complex might be a consequence of JIP1 oligomerization that is known to be mediated by its SH3 to form at least dimers of the signalosome [59].

Next it was determined the incorporation in these JIP1 complexes of different MAP kinases in the absence (Fig. 8C) or presence of VRK2A (Fig. 8D) or VRK2B (Fig. 8E). For this aim the whole cells extracts from Cos1 cells transfected with a mixture of plasmids expressing the different MAP kinases without (Fig. 8C) or with VRK2A (Fig. 8D) or VRK2B (Fig. 8E). The cell extracts were fractionated and complexes at high molecular weigh containing the proteins JIP1, TAK1, TAB1, MKK7 and JNK were detected, suggesting that when the phosphorylation cascade is activated, in this case by TAK1/TAB1 overexpression or by hypoxia stimulation as we recently reported [51], all the MAP kinases tend to be gathered by JIP1 forming an active signalosome. But unexpectedly, the complex had a size of approximately of 1200 kDa (Fig. 8C), which is larger than the expected 340 kDa, probably because the signalosome is an oligomeric complexes formed by several single complexes. However, when VRK2A was included, JNK was the only protein that was mostly free, probably by displacement, although some was also present in the complex (Fig. 8D). A similar complex was isolated with the shorter VRK2B which appeared to be less stable as indicated by the detection of more free individual components and some binary combinations of TAK1 and TAB1 or MKK7 and JNK in their corresponding molecular size fraction (Fig. 8E). We conclude that this complex may constitute the JIP1-JNK signalosome, and depending on the cell context, its protein composition may change, and different protein modulators, as VRK2 in this case, can bind the complex, thus contributing to alter the balance among different or alternative signaling pathways; and for instance the incorporation of VRK2A or VRK2B appeared to destabilize the complex and displace JNK from the signalosome.

### Loss of endogenous VRK2 promotes association of JNK to the signalosome in HeLa cells

**Figure 9:**
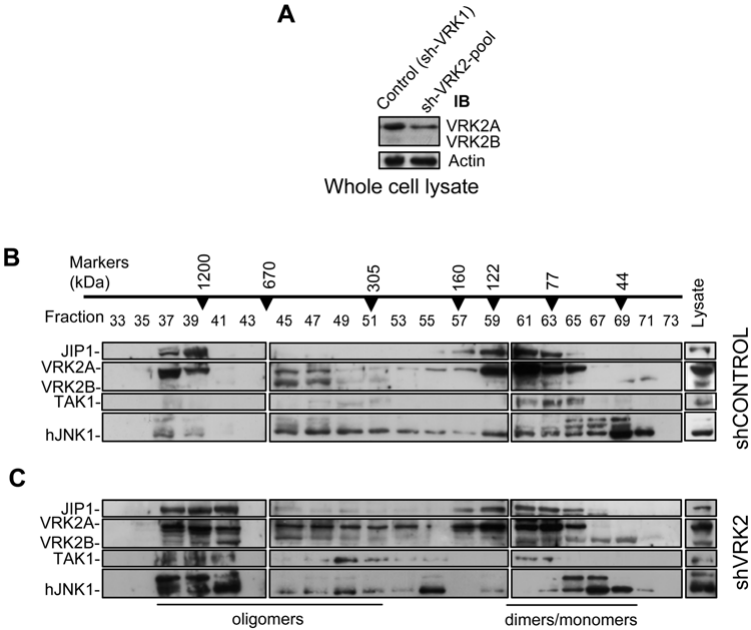
The loss of endogenous VRK2 by shRNA induces an increase in JNK incorporated in the HeLa cells endogenous signalosome. (A). Effect of shRNA on VRK2 endogenous levels in HeLa cells. HeLa cells were transfected as indicated in the methods section with a shControl (pSuperior-shVRK1) or a specific shVRK2 (pSuperior- shVRK2-230+pSuperior-shVRK2-1335) plasmids. The endogenous protein in the whole cell extract was determined in an immunoblot with an anti-VRK2 monoclonal antibody. (B) Fractionation by HPLC and detection of endogenous proteins from HeLa cells transfected with shControl (pSuperior-shVRK1). (C). Fractionation and detection of endogenous proteins from HeLa cells transfected with specific shVRK2 (pSuperior- shVRK2-230+pSuperior-shVRK2-1335) plasmids. All the proteins determined correspond to the endogenous proteins.

Next we tested if reducing the levels of endogenous VRK2 proteins by shRNA could induce an increase in the association of JNK to the signalosome. For this aim HeLa cells, were transfected with pSuperior-shVRK1 as a control or a pool of shRNA specific for VRK2. The effect of these shRNA on the endogenous protein level in whole cell extract is shown in Figure 9A. The extract of these cells were used for fractionation by gel filtration in a Superose 12 10/300 GL column. In the case of the sh-Control most of the JNK protein remained in the small molecular size fractions (Fig. 9B). However in the cells transfected with the specific shVRK2 plasmids, there is a displacement towards the high molecular size fractions of JNK and other proteins of the signalosome including JIP1, TAK1 and VRK2 (Fig. 9C). Notice that this technique does not permit to detect the downregulation of VRK2 expression levels since it separates the proteins by their molecular weight, therefore what is detected is the redistribution of the initial protein present in whole cell extracts (Fig. 9A) in each individual fraction corresponding to a different molecular size. Thus VRK2 protein level increases in the high molecular size fractions while it decreases in the small molecular size fractions. These data are consistent with the interpretation that the incorporation of VRK2 to the signalosome results in a reduction of the JNK protein in the high molecular size complex and thus susceptible of being activated.

### VRK2 downregulates phosphorylation of JIP1-bound JNK

**Figure 10:**
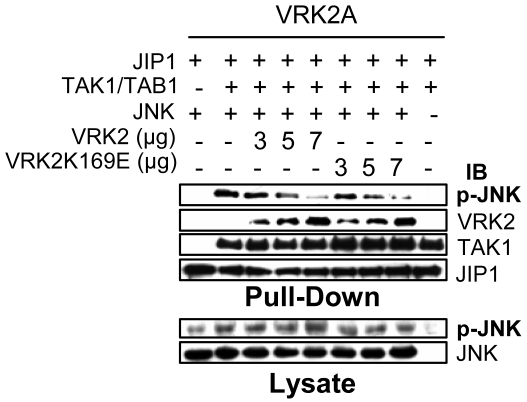
Effect of VRK2 on the activation by phosphorylation of JNK. The proteins were determined in the GST-JIP1 pull-down experiment (top) and in the whole cell lysate (bottom). Cos1 cells were transfected with increasing amounts of plasmids pCEFL-HA-VRK2A and the kinase-dead form (KD: K169E), in the presence of pEBG-GST-JIP1(3 µg), pCMV-HA-TAK1(50 ng) plus de pCMVT-Flag-TAB1(50 ng) and pFlag-JNK(4 µg). The proteins were detected with the corresponding antibody specific for actin, the epitopes GST, HA or Flag, and for the specific phosphorylation in Thr183 and Tyr185 of JNK (p-JNK).

The consequence of a displacement of JNK from the signalosome containing VRK2A is the interference with signal transduction by this pathway that might be reflected in a reduction of the JNK activation. Functionally the interaction of VRK2 with JIP1, and the first two kinases (TAK1 and MKK7), might affect the transmission of the signal through this signaling complex, and this may be manifested as a change in the activation of JNK. In order to activate JNK by phosphorylation it is necessary to receive an activating signal from an upstream MAPKKK, and for this purpose TAK1 was overexpressed, together with TAB1 [49], since TAK1 activation promotes JIP1-JNK association [51] and JNK activation by phosphorylation on Thr183 and Tyr185 [60], [61]. To ascertain the effect of VRK2, Cos1 cells were transfected with increasing amounts of both VRK2A and VRK2B isoforms, or their kinase-dead variants VRK2 (K169E) in the presence of the activating TAK1/TAB1, JIP1 and JNK. Then, the whole lysates were used for a pull-down experiment to bring down the proteins associated to GST-JIP1, and identified in an immunoblot (Fig. 10). In the pulled-down proteins, as VRK2A protein (or its kinase-dead variant) increased, the level of phosphorylated JNK clearly decreased, a difference that was not detectable in the whole cell lysate, and thus is likely to represent a subpopulation of JNK bound to JIP1. VRK2B also decreases the level of JNK phosphorylation, but less noticeable (not shown), effect that was also observed in the downregulation of AP1 dependent transcription in response to IL-1β (Fig. 2A and B), suggesting that VRK2A isoform downregulates IL-1β signal more efficiently and specifically than VRK2B. Therefore, functionally, the association of VRK2A or VRK2B to the JIP1-MAP kinase signaling complex should reduce JNK phosphorylation, because the complex formed by VRK2-JIP1 can not interact with JNK by forming an alternative signalosome. The consequence of this alternative complex formation should be a reduction in c-Jun dependent transcription.

## Discussion

Modularity in the components of a signaling pathway permits a large flexibility by interaction with proteins that can play a regulatory role of its activity, or alter the balance between alternative signal response pathways. In this report we have characterized how a new protein, VRK2, by interacting with the JIP1 scaffold protein that assembles MAP kinase signaling complex, is able to restrict the flow of the signal in response to IL-1β, mediated by TAK1, or other stimuli such as hypoxia, which has been recently observed in our laboratory to activate JNK through this signalosome [51]. JIP1 by itself is able to form a large oligomeric complex or signalosome, upon which other components, such as regulators of the pathway can be assembled, for example VRK2A or VRK2B. The JIP1-VRK2 interaction is also supported by colocalization studies that demonstrate that the patterns of JIP1 and VRK2A immunostaining are overlapping (Fig. 5).

The VRK2 interaction occurs through the C-terminal region of the JIP1 molecule which appears to alter the proportions of MAP kinases associated with JIP1. Furthermore the isolation of large protein complexes that include several kinases indicates that any effect of VRK2 on signaling might be by modulating the whole complex. It is important to draw the attention to the fact that the downregulation of signaling is a consequence of the protein interaction, and not of the kinase activity. However, the precise mechanism by which this occurs is probably mediated by the direct interaction of VRK2 with JIP1 that destabilizes the complex and reduce its binding to JNK, thus locking JIP1 in a conformation that interrupts the transduction of the signal through the complex, which was detected as a decrease in the phosphorylation of JNK complexed with JIP1 (Fig. 10) and the downregulation of AP1 transcriptional activation triggered by IL-1β and hypoxia [51], in the presence of both VRK2 isoforms. JNK is a minor component of the signalosome and is mostly free in the cell before or even after stimulation (Fig. 8, 9), thus its interaction with the complex might be transient since once activated it should be translocated into the nucleus to phosphorylate transcription factors. The direct interaction of VRK2 with JIP1, TAK1 and MKK7, but not JNK, can block the conformational change needed to prevent incorporation of JNK to the complex (Fig. 8D,E). On the other hand, the downregulation of endogenous VRK2 protein levels stabilizes all the components of the signalosome (Fig. 9), indicating again that VRK2 protein levels modulate the signalosome stability and therefore the signaling through the pathway.

Alternatively, the decrease of JNK phosphorylation raises the possibility that a phosphatase activity could be required suggesting that VRK2 association might gather some MAPK phosphatases such as the dual-specificity phosphatases MPK7 or M3/6, which have been described to bind to JIP1 and reduce JNK activation, but this requires bound JNK [62], which appears to be reduced by VRK2A. This alternative mechanism is implicated in the downregulation of ERK activity by the VRK3, a distant relative of VRK2 [29], [39], by a mechanism independent of its kinase activity. VRK3 has been shown to bind vaccinia H1-related (VHR), which specifically dephosphorylate and inactivates ERK [41], suggesting a conserved role of VRK proteins in the downregulation of MAPK activity although by different mechanisms. But VRK2 does not interact with VHR phosphatase, probably because of their sequence divergence in the interaction region.

Other proteins interacting with JIP1 also modulate its signal transmission. It has been also demonstrated that Akt suppresses JNK activity by a mechanism independent of its kinase activity, through direct binding to the C-terminal of JIP1 (residues 471-660), similar to that interacting with VRK2. The Akt1-JIP1 interaction reduces the JIP1-JNK interaction, and downregulates JNK activation [22], [63], and thus promotes an upregulation of Akt survival signal [64]; this mechanism is similar to that mediated by VRK2A. Notch also interacts with JIP1, by its JBD region, and thus competes with binding to JNK, resulting in downregulation of JNK activation [65]. In all these interactions the common functional consequence is a reduction of JNK activation. The interaction of VRK2 proteins with JIP1 complexes and the downregulation of IL-1β signaling can have wide biological implications. The JNK complexed with JIP1 responds to specific stimulation such as oxidative stress [21], [22] or IL-1β but not to UV radiation or anisomicyn [20]. In addition it has been demonstrated that JIP1 content in β-cells is a crucial regulator of JNK signaling pathway and of cytokine-induced apoptosis [1]. The level of VRK2 can modulate the response to IL-1β by regulating the flow through the JIP1-JNK complex, and perhaps alter the balance between different signaling pathways for those molecules that have several response routes, as is the case for IL-1β.

Regarding the biological consequences, the innate immune responses are mediated by a group of receptors that belong to the Toll-like/IL-1R [66] and TNFR families in response to a variety of cytokines [67] or infections [68]. Furthermore, IL-1β is implicated in multiple pathologies leading to tissue damage such as arthritis, transplant rejection, Alzheimer's, Crohn's disease, systemic lupus erythematosus, septic shock, tumorigenesis and metastasis, lymphoprolipherative disorders and pulmonary fibrosis [67]. Therefore VRK2 levels might alter the balance between alternative signaling pathways

**Figure 11:**
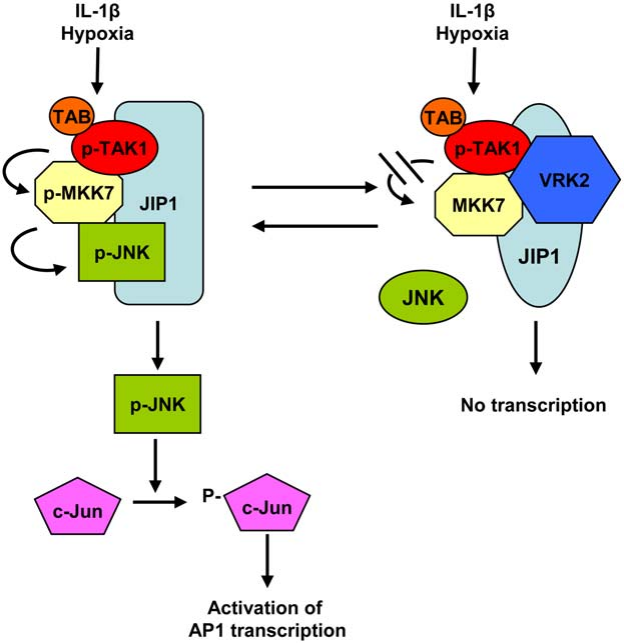
Model of the two alternative signalosomes assembled on JIP1. The signalosome that does not contain VRK2 proteins (left) allows signal transmission initiated in the MAPKKK (TAK1) and reached till activation of transcription. The signalosome containing VRK2 proteins that interact with both TAK1 and JIP1 has a different conformation and the signal is blocked and ca not be transmitted. This conformation does not interact with JNK. The two alternative conformations might be in different proportions in the cell depending on the relative concentration of their components as well as their subcellular localization.

The different response to interleukin-1β stimulation (this report) or hypoxia [51] can be explained if there are two alternative conformations of JIP1, one containing VRK2 protein and the other not (Fig. 11). The JIP1 signalosome without VRK2 allows signal transmission and subsequent activation of transcription. The signalosome containing VRK2 prevents transmission of the signal, detected as a reduction in phosphorylation of intermediate steps, and also by the loss of JNK incorporation into the complex, thus the signal can not be transmitted.

In this work it has been shown that VRK2 downregulates the response to IL-1β mediated via TAK1, thus the response to IL-1β may be relatively enhanced by altering the balance between the pathways responding to multiple simultaneous stimuli the cell is exposed in its tissue niche. This type of situation can in part help to explain why many cellular responses to a common stimulation have apparently contradictory biological effects, for example proliferation versus cell-cycle arrest. Levels and subcellular localization of VRK2 may modulate different responses where the assembly of JIP1 complexes is required, since in its presence the level of activation will be lower the higher the level of VRK2 protein is. Thus the combination of the proteins expressed, namely VRK2 or additional proteins that remain to be identified, their levels, and perhaps subcellular localization can play an important role in determining the balance among the different pathways in response to a common stimulation.

## Materials and Methods

### Plasmids and reagents

The prokaryotic pGEX-JIP1 (1-127), pGEX-JIP1 (128-282) and pGEX-JIP1 (283-660) and the mammalian expression plasmids pEBG-GST-JIP1 and all its deletion variants were from R. Davis [20]. Plasmid pCMV5-Flag-MKK7β1 was from A. Whitmarsh [62]. Plasmids expressing pHA-TAK1 wild type and pFlag-TAB1 were from K. Matsumoto [69]. Plasmid pFlag-JNK was made by PCR from plasmid pHA-JNK from S. Gutkind (NIH, Bethesda, MD); the prokaryotic expression construct in pGEX-4T: GST-VRK2A and GST-VRK2B, and mammalian expression construct pCEFL-HA-VRK2A and pCEFL-HA-VRK2B had been previously reported [40]. The pAP1-Luc reporter was from Stratagene (San Diego, CA). Plasmid pRL-tk from Promega Biotech (Madison, WI) was used for internal control in luciferase assays. The clones for shRNA were made in plasmid pSuperior-Retro-puro following manufacturer instructions (Oligoengine, Seattle, WA). The sequence of the plasmid p-sh-RNA-VRK1 was previously described [31]. The plasmids p-sh-RNA-VRK2-230; p-sh-RNA-VRK2-438 and p-sh-RNA-VRK2-1335 were previously described [40].

### In vitro interaction between GST-JIP1 fusion proteins and VRK2A transcribed-translated in vitro

The plasmids encoding the GST-JIP1 fusion proteins, pGEX-JIP1 (1-127), pGEX-JIP1 (128-282) and pGEX-JIP1 (283-660) were expressed in BL21DE3, then purified with glutathione Sepharose beads (GE Healthcare) and eluted from them with glutathione according to the manufacturer instructions. The VRK2A protein was transcribed and translated in the presence of [^35^S]-methionine using a reticulocyte lysate in vitro transcription-translation kit (Promega Biotech, Madison, WI) according to the manufacturer's instructions. Briefly, 2 µg of each GST-JIP1 fusion protein or GST protein, as a control, were incubated at 4°C with ^35^S-labeled VRK2A in 200 µl of the binding-washing buffer (50 mM Tris, pH 7.4, 250 mM NaCl, 0.1% Triton X-100, 5 mM EDTA, 2 mM DTT) for 2 h. The complexes analyzed by SDS-polyacrylamide gel electrophoresis. The gels were transferred to an Immobilon-P membrane (Millipore) and the membrane exposed to X-ray films or analyzed with a Bio-Rad phosphorimager to detect the ^35^S-labeled VRK2A. The GST-JIP precipitated proteins were detected by membrane staining.

### Cell culture, transfections and immunoblots

Cos1 and HeLa cells were grown in DMEM supplemented with 10% fetal calf serum, antibiotics in 5% CO_2_ humidified atmosphere. For assays of transcriptional activity using a pAP1-Luc reporter plasmid, Cos1 cells were plated in 35 mm-diameters dishes and transfected with 0.8 µg of synthetic reporter plasmid pAP1-Luc or 10 ng of pRL-tk, and the indicated among of the specific kinase constructs or shRNA expressing plasmid specified in each experiment. The total DNA was mixed with 6 µl of JetPEI transfection reagent (Polytransfection, Ilkirch, France). 4 hours after transfection media was changed for serum-free DMEM and cells were treated during 6 hours with 10 ng/ml of IL-1β (Preprotech, London, UK). Cells were lysed 48 hours after transfection and luciferase activity determined with a Dual-Luciferase reporter reagent from Promega.

For immunoblot analysis, cells were harvested 48 hours post-transfection and lysed with buffer containing in 20 mM Tris-HCl pH 7.4, 137 mM NaCl, 2 mM EDTA, 25 mM β-glycerophosphate, 10% (v/v) glycerol and 1% Triton-X100 with inhibitors of proteases and phosphatases (1 mM PMSF, 10 mg/ml aprotinin, 10 mg/ml leupeptin, 1 mM Na orthovanadate). 50 µg of total protein lysate were fractionated in a 10% SDS-polyacrylamide gel and analyzed by western blot to identify the proteins present depending on the experiment.

### VRK2 knock-down by shRNA

For assays of transcriptional activity using shRNA specific for VRK2, HeLa cells were plated in 35 mm-diameters dishes and transfected with 0.2 µg of pAP1-Luc, 10 ng of pRL-tk and 6 µg of the specific shRNA expressing plasmids indicated previously. The total DNA was mixed with 12 µl of JetPEI transfection reagent. Cells were treated as indicated before and luciferase activity was determined 48 hours post-transfection with a Dual-Luciferase reporter reagent from Promega.

### JIP1 knock-down by siRNA interference

HP Validated siRNA duplexes for JIP1 were purchased from Quiagen (Valencia, CA). The targeted sequences for JIP1 (gene accession number NM_005456) were TGGCATCAGCTTACAGTGCAA (siRNA JIP1#1) and CTGGAGGAGTTTGAGGATGAA (siRNA JIP1#2). A functional siCONTROL nontargeting siRNA pool from Dharmacon was used as a negative control, and fluorescently labeled siGLO Lamin A/C siRNA was used for lamin silencing and transfection efficiency. HeLa cells were plated in 35 mm-diameters dishes and transfected with 100 pmols of siRNAs using 10 µl of Lipofectamine^TM^ 2000 transfection reagent (Invitrogen); 24 hours later, cells were retransfected with 0.2 µg of pAP1-Luc and 10 ng of pRL-tk using Lipofectamine ^TM^ 2000 transfection reagent. Cells were treated as indicated before and luciferase activity was determined 48 hours post-transfection with a Dual-Luciferase reporter reagent from Promega.

### Detection of protein complexes by pull-down experiments and gel filtration chromatography

For pull-down experiments Cos1 cells grown in 100 mm dishes were transfected with different fragments of fusion proteins in mammalian expression vectors. The amount and type of the specific plasmid is indicated in each individual experiment. Whole cell extracts prepared 48 hours after transfection were lysed in the buffer mentioned before. To bring down the fusion protein with its associated proteins the extract was mixed with glutathione-Sepharose beads (GE Healthcare) for 12 hours at 4°C with gentle shaking. The washed beads were loaded in a SDS-PAGE gel and transferred to an Immobilon-P membrane (Millipore) and the western blot was analyzed for the indicated proteins with the corresponding antibody in individual experiments. For isolation of protein complexes by gel filtration chromatography Cos1 cells were transfected with 3 µg of pGST-JIP1, 50 ng of pHA-TAK1, 50 ng of pFlag-TAB1, 0.2 µg of pFlag-MKK7, 4 µg of pFlag-JNK and 4 µg of pCEFL-HA-VRK2A or pCEFL-HA-VRK2B. 48 hours later protein extracts were prepared using buffer containing in 20 mM Tris-HCl pH 7.4, 137 mM NaCl, 2 mM EDTA, 25 mM β-glycerophosphate, 10% (v/v) glycerol and 1% Triton-X100 with inhibitors of proteases and phosphatases (1 mM PMSF, 10 mg/ml aprotinin, 10 mg/ml leupeptin, 1 mM Na orthovanadate). Insoluble material was removed by centrifugation at 16,000×g for 20 min. The supernatant, containing 1.5 mg of dissolved protein, was fractionated by HPLC gel filtration through a Superose 12 10/300 GL column (GE Healthcare). HPLC was performed with an HP 1100 model from Agilent Technologies (Germany) equipped with a ChemStation software, and developed with a buffer containing 50 mM Tris-HCl, pH 7.5, 1 mM EDTA, 100 mM KCl at a flow rate of 0.1ml/min. 0,2 ml-fractions were collected, precipitated and resolved on a 7,5 or 10% polyacrylamide gel and immunoblotted. Molecular weight markers used to calibrate the column were: bovine thyroglobulin (670000), apoferritin from horse spleen (440000), alcohol dehydrogenase from yeast (150 000), bovine serum albumin (66000) and bovine carbonic anhydrase (29000), all from Sigma. The effluent was monitored at 280 nm.

### Detection of endogenous JIP RNA

The expression of four endogenous human JIP genes was determined by real time quantitative RT-PCR was performed as previously described [40].Total RNA was extracted using the “RNeasy extraction kit” from Quiagen (Hilden, Germany). RNA was analyzed and quantified using a Bioanalyzer 2100 nano-lab chip from Agilent Technologies (Germany). 100 ng of total RNA were used in a one-step reverse transcription real-time PCR amplification reaction using the “Quantitec SYBR Green RT-PCR kit” from Quiagen in an iCycler equipped with an iCycler iQ5 Software (BioRad, Hercules, CA). The RT step was performed at 50°C for 30 minutes, and inactivated at 95°C for 30 seconds, the PCR phase consisted of one cycle at 95°C for 15 minutes followed by 50 cycles with three steps, of 94°C for 15 seconds, 58°C for 30 seconds and 72°C for 1 minute. PCR products were resolved in a 2% agarose ethidium-bromide gel. The primers used for JIP1 amplification were for JIP1(forward: 5′-TCAGTCCAGGTTCCCTATCAC-3′; reverse: 5′-TTGACGCCTATCTTCACACC-3′), JIP2 (forward: 5′-GCTTTTCCTCAGATCCGTTC-3′, reverse: 5′-CACTTGGAAGCCGACATTAC-3′), JIP3 (forward: 5′-AAGCCTCTATCCTGTCTGTC-3′; reverse: 5′-CCTCCAAGGTGAGTCTTCTG-3′), JIP4 (forward: 5′-AGCCCACAAAGTAGCAGTAG-3′; reverse: 5′-GACAGAAGGTTCAAGTGGAAG-3′), and for GAPDH (forward: 5′-GGTCTTACTCCTTGGAGGCCATGT-3′; reverse: 5′-ACCTAACTACATGGTTTACATGTT-3′).

### Antibodies and reagents

Human VRK2 was detected with a rabbit polyclonal antibody [40]. Human JNK1 was detected with monoclonal (G151-333) from BD Pharmingen. Human JIP1 protein was detected with rabbit polyclonal (M-300) antibody; calnexin was detected with a monoclonal (AF18); JNK phosphorylated in Thr183 and Tyr185 was detected with a monoclonal antibody (G7); endogenous TAK1 was detected with monoclonal (C9); and GST protein was detected with a monoclonal (B-14), all from Santa Cruz. The HA epitope was detected with a monoclonal (HA.11) from Covance (Berkeley, CA). The FLAG epitope was detected with a rabbit polyclonal antibody from Sigma. Actin was determined with a monoclonal antibody (clone AC-15) from Sigma. A goat HRP-anti mouse antibody was from GE Healthcare. A sheep HRP anti-rabbit antibody was from Sigma. FluorolinkCy2 anti rabbit IgG, FluorolinkCy3 anti rabbit IgG and FluorolinkCy2 anti mouse IgG were from GE Healthcare. Mitochondria were detected using the MitoTracker Red CMXRos reagent (Molecular Probes, Invitrogen). Recombinant human IL-1β was from Peprotech (London, UK).

The JIP1 region recognized by the anti-JIP1 rabbit polyclonal antibody (M-300) was tested using 100 ng of the GST-fusion proteins GST-JIP1 1-127, GST-JIP1 127-282, GST-JIP1 283-660 and GST that were subjected to immunoblot analysis with a GST specific monoclonal antibody and the αJIP1 antibody. To test the specificity of the anti JIP1 antibody, an aliquot of the diluted antibody was incubated overnight at 4°C with 2 µg of GST-JIP1 (1-127) fusion protein, and as a control, another aliquot of the diluted antibody was incubated with 2 µg of GST fusion protein. The two aliquots were used to perform an immunoblot with HeLa and Cos1 cell extracts to detect the endogenous JIP1 protein.

### Confocal microscopy

The subcellular localization of JIP1, VRK2 endogenous or transfected proteins were determined in the indicated cells lines grown on coverslips and stained with the corresponding antibodies. Cells were seeded in 60 mm dishes and transfected 24 hours later with 5 µg of pCEFL-HA-VRK2A and B mixed with 10 µl of JetPEI transfection reagent (Polytransfection, Ilkirch, France). 48 hours post-transfection the slides were collected and fixed with 3% paraformaldehyde for 30 minutes at room temperature, then treated with 100 mM glycine for 10 min at room temperature and then permeabilized with 0.2% Triton X-100 for 30 min at room temperature. The cells were blocked with 1% BSA in PBS for 30 min at room temperature followed by a double immunostaining with the corresponding antibodies. Finally cells were stained with DAPI (4′, 6′-diamidino-2-phenylindole) (Sigma) 1∶1000 in PBS for 10 min at room temperature, then cells were washed with PBS, and slides were mounted with Gelvatol (Monsanto). The images were acquired with a Zeiss LSM510 confocal microscope and the analysis was performed with the LSM Image Examiner program (Zeiss).
